# Decolonising the ‘red’ reflex test: transitioning from terminology based on colour to anatomy

**DOI:** 10.1038/s41433-024-03433-2

**Published:** 2024-11-21

**Authors:** Cieren Kelly, Martin Anderson, Harry Smith, Obaid Kousha, Nicki Hitchcott, M. Ashwin Reddy, Andrew Blaikie

**Affiliations:** 1 Global Health Team, School of Medicine Medical and Biological Sciences Building, University of St Andrews, North Haugh, St Andrews, KY16 9TF UK; 2 School of Computer Science, University of St Andrews, Jack Cole Building, North Haugh, St Andrews, KY169SX UK; 3 School of Modern Languages, University of St Andrews, Buchanan Building, Union Street, St Andrews, KY16 9PH UK; 4 Queen Mary University of London & Retinoblastoma Service, Royal London Hospital, Barts Health NHS Trust, London, E1 1BB UK

**Keywords:** Education, Diagnosis

We wish to bring attention to an important issue in ophthalmology: the need to decolonise and update the terminology of the ‘red’ reflex test. This essential yet simple examination, used to detect life and sight-threatening eye conditions in newborns and infants [1], is currently named and understood through a Eurocentric lens, which does not reflect the global population it serves.

The ‘red’ reflex test, part of the Newborn and Infant Physical Examination (NIPE) in the UK and recommended by the World Health Organization for routine postnatal care, involves observing the reflection of light from the fundus of both eyes using a direct ophthalmoscope [2, 3]. An abnormal result is indicated by differences in colour or brightness between the eyes, absence of the reflex, or an atypical colour for a particular ethnic group.

The test’s name implies that a ‘normal’ result produces a red reflex, which is typically seen in individuals who self-identify as white. However, it is recognised that the reflex appears differently across ethnic groups. The NIPE guidelines acknowledge this variation, noting that the reflex can be less bright and appear magnolia in colour in Black, Asian, or minority ethnic babies [3].

This discrepancy between the test’s name and its appearance in diverse populations creates several problems:

- Misinterpretation and over-referral: Non-white babies are often unnecessarily referred for urgent second opinions, wasting resources and causing undue anxiety for families.
- Training challenges: The misnomer creates confusion when training healthcare professionals, particularly in non-white majority countries.
- Perpetuation of biases: The continued use of this terminology reinforces long-standing historical biases in medical practice.

To address these issues, we conducted an exploratory study to gather objective and subjective evidence of the ‘normal’ appearance of this test across different ethnic groups. We captured reflex videos of six adults with diverse self-identified ethnicities (Black, Asian Indian, Asian Afghan, Southeast Asian, White with fair hair, and White with dark hair) using an Arclight ophthalmoscope attached to a smartphone camera (Fig. 1).

**Fig. 1:**
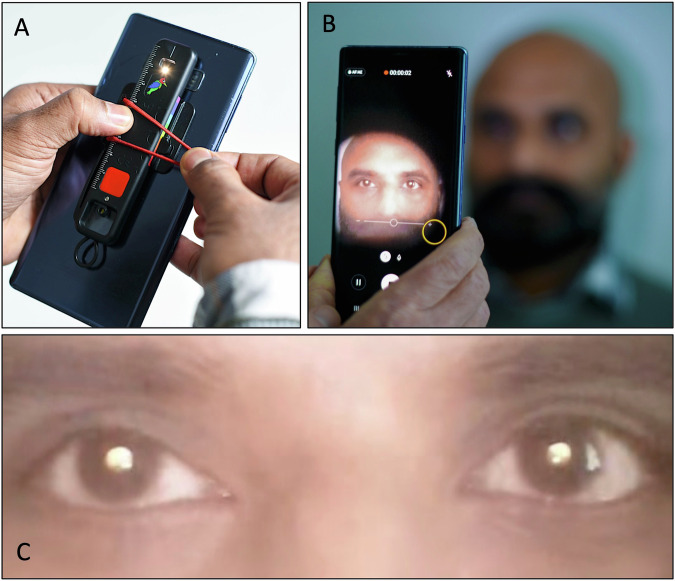
Acquisition of fundal reflex videos using an Arclight ophthalmoscope and a smartphone. **A** Attach Arclight direct ophthalmoscope to camera of phone. **B** Acquire reflex video in a darkened room. **C** Subjective (video review by naïve participants) and objective (RBG score of still image pupil space) analysis.

Twenty-seven naïve observers (first-year medical students) recorded their subjective impressions of the colour seen in the pupil space for each video. Additionally, we used an algorithm to calculate the mean objective Red/Green/Blue (RGB) value of all ‘pupil space’ pixels, converting this to a Hexadecimal code and associated colour.

Our results corroborated widely held opinions: subjectively, yellow, orange, and white were the most commonly recorded colours, while objectively, brown and orange were the most frequent (Table 1). These findings provide the first empirical evidence supporting the need to rename the ‘red’ reflex test.

**Table 1:**
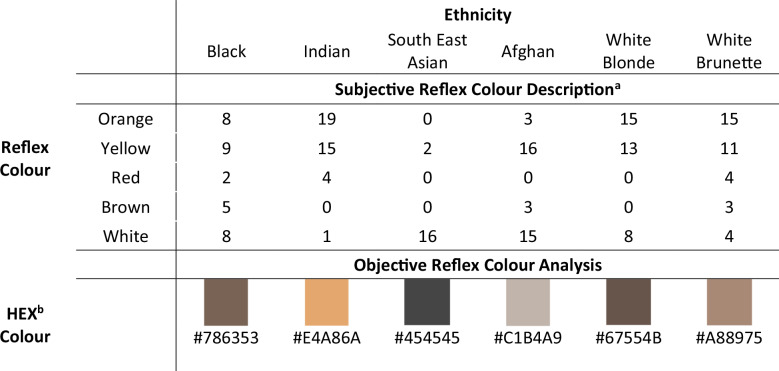
Summary of subjective colour description and objective algorithmic colour analysis of fundal reflex videos.

^a^Based on 27 naïve observers describing subjective colour perception of pupil space during fundal reflex videos.

^b^HEX: Hexadecimal code (based on algorithmic analysis of red/blue/green composition of pupil space in video frames) and the colour associated with that code.

We propose changing the terminology to the “Fundal Reflex Test,” an anatomically accurate description that avoids implying a specific colour, thereby reducing misunderstanding and promoting equity and inclusivity. Decolonising medical terminology is essential for ensuring accurate diagnoses and culturally competent care.

Renaming the ‘red’ reflex test is an important step toward addressing historical biases in ophthalmology. We urge the broader ophthalmology community to adopt this new terminology and continue challenging other areas where such biases may persist, ensuring that eye care becomes more inclusive worldwide.

## Data Availability

The datasets generated during and analysed during the current study are available from the corresponding author upon reasonable request.
